# A *Drosophila* model of the neurological symptoms in *Mpv17*-related diseases

**DOI:** 10.1038/s41598-022-27329-x

**Published:** 2022-12-31

**Authors:** Atsushi Kodani, Mizuki Yamaguchi, Ririka Itoh, Man Anh Huynh, Hideki Yoshida

**Affiliations:** 1 Department of Applied Biology, Kyoto Institute of Technology, Matsugasaki, Sakyo-Ku, Kyoto, 606-8585 Japan; 2 Advanced Insect Research Promotion Center, Kyoto Institute of Technology, Matsugasaki, Sakyo-Ku, Kyoto, 606-8585 Japan

**Keywords:** Disease model, Diseases of the nervous system

## Abstract

Mutations in the *Mpv17* gene are responsible for *MPV17*-related hepatocerebral mitochondrial DNA depletion syndrome and Charcot–Marie–Tooth (CMT) disease. Although several models including mouse, zebrafish, and cultured human cells, have been developed, the models do not show any neurological defects, which are often observed in patients. Therefore, we knocked down *CG11077* (*Drosophila Mpv17*; *dMpv17*), an ortholog of human *MPV17*, in the nervous system in *Drosophila melanogaster* and investigated the behavioral and cellular phenotypes. The resulting *dMpv17* knockdown larvae showed impaired locomotor activity and learning ability consistent with mitochondrial defects suggested by the reductions in mitochondrial DNA and ATP production and the increases in the levels of lactate and reactive oxygen species. Furthermore, an abnormal morphology of the neuromuscular junction, at the presynaptic terminal, was observed in *dMpv17* knockdown larvae. These results reproduce well the symptoms of human diseases and partially reproduce the phenotypes of *Mpv17-*deficient model organisms. Therefore, we suggest that neuron-specific *dMpv17* knockdown in *Drosophila* is a useful model for investigation of *MPV17*-related hepatocerebral mitochondrial DNA depletion syndrome and CMT caused by *Mpv17* dysfunction.

## Introduction

Human MPV17 is a putative channel-forming protein of the inner mitochondrial membrane and it is involved in mitochondrial deoxynucleotide homeostasis^1–3^. *MPV17* mRNA is widely expressed in tissues, including the liver, kidney, muscle, lung, brain, and heart (https://www.proteinatlas.org/ENSG00000115204-MPV17, Human Protein Atlas proteinatlas.org). Autosomal recessive mutations in the *MPV17* gene cause *MPV17*-related hepatocerebral mitochondrial DNA depletion syndrome (MDDS), including Navajo neurohepatopathy, which typically has hepatic and gastrointestinal manifestations, metabolic derangements, and neurological involvement, including developmental delay, hypotonia, microcephaly, and motor and sensory peripheral neuropathy; most patients die by childhood^4^. Patients with Navajo neurohepatopathy show more severe symptoms and often die in infancy or early childhood due to progressive liver dysfunction associated with depletion of mitochondrial DNA (mtDNA)^5^. Autosomal recessive mutations in the *MPV17* gene have also been identified in patients with Charcot-Marie-Tooth (CMT) disease, the most common inherited motor and sensory peripheral neuropathy^6–8^.

To elucidate the mechanism of the pathogenesis of *MPV17*-related hepatocerebral MDDS, model organisms such as *MPV17* knockout (KO) mice and KO zebrafish have been established and analyzed. *MPV17* KO mice show significantly reduced longevity, lower body weight, and hair graying, with defects in the kidney, skin, and peripheral nervous system^9–12^. *MPV17* deficiency in mice leads to a reduced deoxynucleotide pool resulting in mtDNA depletion in the liver, but not in the kidney or brain^2^. In zebrafish, diverse phenotypes have been reported in two *mpv17* mutants (*roy orbison* (*roy*) and *transparent* (*tra*)), and *Mpv17* KO zebrafish generated by the CRISPR/Cas9 system^13–17^. While *roy* and *tra* mutants show only transparent phenotypes with a loss or strong reduction of iridophores and melanophores, *Mpv17* KO zebrafish show reduced longevity and defects in muscle, liver, and energy supply during development in addition to a strong reduction of iridophores and melanophores^13–17^. However, in these studies, the neurological symptoms have not been fully analyzed, albeit human patients with *MPV17*-related hepatocerebral MDDS commonly show them. *Drosophila* models have been successfully used to study various neurological disorders^18–22^. In the present study, we establish a *Drosophila* model to analyze the neurological pathogenesis of *MPV17*-related hepatocerebral MDDS.

## Results

### *CG11077* is a *Drosophila* ortholog of human *MPV17*

**Figure 1:**
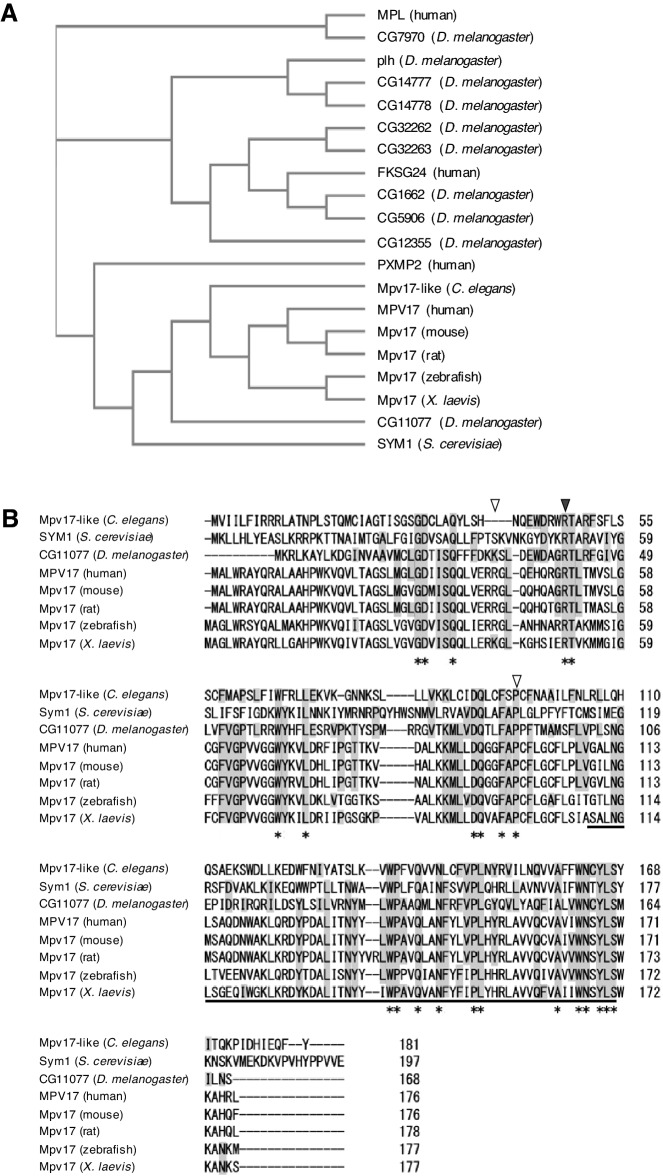
CG11077 is an ortholog of human MPV17. (**A**) A phylogenetic tree was constructed with amino acid sequences of MPV17/PMP22 family proteins, human (MPL, FKSG24, PXMP22, and MPV17), *S. cerevisiae* SYM1, *D. melanogaster* (CG7970, CG14777, CG144778, CG32262, CG32263, CG1662, CG12355, and CG11077), *C. elegans* Mpv17-like, and Mpv17s (mouse, rat, zebrafish, and *X. laevis*). The sequence similarity of MPV17/PMP22 family proteins among model organisms and the human MPV17 family was analyzed. (**B**) Multiple alignment of amino acid sequences of Mpv17 among model organisms and human, including CG11077, was carried out with the Clustal Omega multiple sequence alignment program. Amino acids conserved among all organisms are marked by asterisks and those conserved between *D. melanogaster* and others are highlighted in gray. The black and white arrowheads indicate the amino acids mutated in *MPV17*-related hepatocerebral MDDS (R50Q) and CMT (R41Q and P98L) patients, respectively. Underlining indicates the amino acid sequences conserved between PMP22 and MPV17 (Mpv17/PMP22: IPR007248).

In the *Drosophila* genome, there are ten MPV17/PMP22 family genes, including nine uncharacterized genes named with CG numbers. To identify the *Drosophila* ortholog of human MPV17, the amino acid sequence similarity of MPV17/PMP22 family proteins among model organisms and the human MPV17 family was analyzed (Fig. 1A). Because the phylogenetic tree clearly shows that CG11077 is the best candidate for a *Drosophila* ortholog of human MPV17, we named *CG11077 Drosophila Mpv17* (*dMpv17*). The alignment of Mpv17 proteins among model organisms indicates that these proteins, including the MPV17/PMP22 domain marked by underlining, are well conserved (Fig. 1B). The mutations identified in patients with *MPV17*-related hepatocerebral MDDS (R50Q)^4^ and CMT (P98L, R41Q)^6–8^, which both show peripheral neuropathy, are identical or similar between *Drosophila* and humans (Fig. 1B). Because these mutations have been identified as recessive mutations in patients and predicted to be hypomorphic mutations^6,8^, we knocked down *dMpv17* specifically in the nervous system during *Drosophila* development to elucidate the effect of *dMpv17* depletion on nerve cells.

### Phenotypes of neuron-specific *dMpv17* knockdown flies

**Figure 2:**
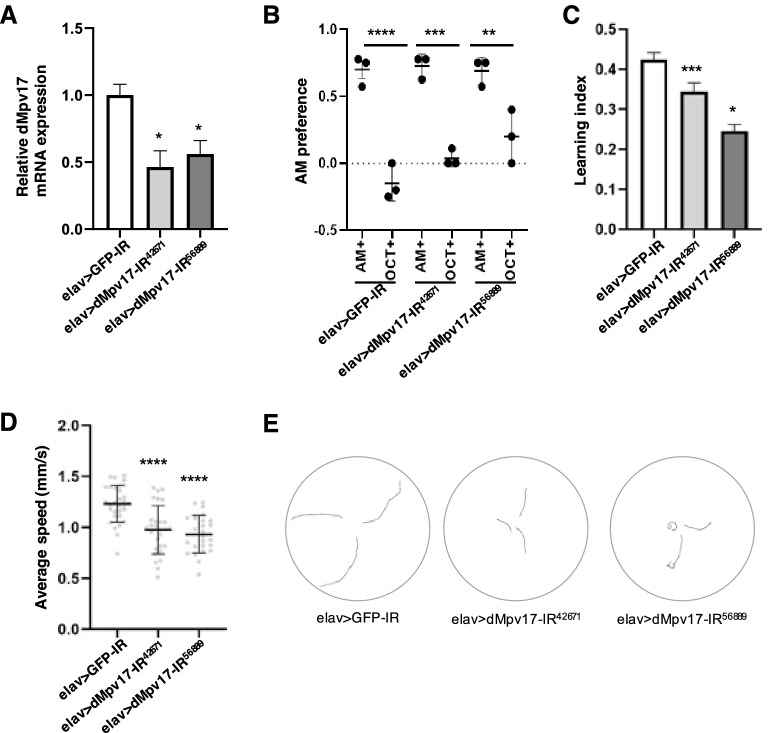
Neuron-specific *dMpv17* knockdown larvae show neurological-defect phenotypes. (**A**) Total RNA was extracted from the CNS of third instar larvae carrying elav > GFP-IR (*UAS-GFP-IR/*+*; elav-GAL4/*+*;*+), elav > dMpv17-IR^42671^ (*elav-GAL4/UAS-dMpv17-IR*^*42671*^*;*+), and elav > dMpv17-IR^56889^ (*elav-GAL4/UAS-dMpv17-IR*^*56889*^*;*+). The mRNA levels of *dMpv17* and *G6PD* in each strain were detected by qRT-PCR. *G6PD* was used as an internal control. ***P < 0.01, statistical analysis was performed using one-way ANOVA followed by Dunnett’s multiple comparisons test vs elav > GFP-IR. *n* = 3. (**B**, **C**) The learning ability of larvae carrying elav > GFP-IR, elav > dMpv17-IR^42671^, and elav > dMpv17-IR^56889^ was tested in the odor-taste learning assay. AM preference (**B**) and larval learning index (**C**) were quantified. *P < 0.05, ***P < 0.001, using one-way ANOVA followed by Dunnett’s multiple comparisons test vs elav > GFP-IR. *n* = 3. (**D**, **E**) Larval locomotor ability was tested in the crawling assay with elav > GFP-IR, elav > dMpv17-IR^42671^, and elav > dMpv17-IR^56889^. (**D**) The average crawling speed was quantified. Statistical analysis was performed using one-way ANOVA followed by Dunnett’s multiple comparisons test vs elav > GFP-IR. *n* = 25–31. (**E**) Typical trajectories of larvae with each genotype.

To investigate the effect of *dMpv17* depletion on nerve cells, we knocked down *dMpv17* in the nervous system by using a pan-neuronal GAL4 driver, *embryonic lethal abnormal vision* (*elav*)-GAL4. The knockdown efficiency of *dMpv17* mRNA in the larval central nervous system (CNS) of two independent knockdown lines (elav > dMpv17-IR^42671^ and elav > dMpv17-IR^56889^) was determined by RT-qPCR. *dMpv17* mRNA levels were reduced to 46% and 54%, respectively, in elav > dMpv17-IR^42671^ and elav > dMpv17-IR^56889^ compared to the control elav > GFP-IR (Fig. 2A). We thus confirmed the effective knockdown of *dMpv17* in two independent RNAi lines. With these lines, we examined whether *dMpv17* knockdown flies showed neurological defects, such as peripheral neuropathy, seizures, or cognitive disability, that correspond to neurological symptoms of human *MPV17*-related MDDS.

Because *MPV17*-related MDDS patients often show cognitive impairment^4^, we investigated the learning ability of *dMpv17* knockdown larvae by testing whether the larvae could learn the relationship between reward and odor. Whereas *n*-amyl acetate (AM), a compound with a fruity odor, was still attractive to *dMpv17* knockdown larvae, as it is to control larvae (Fig. 2B), the larvae of both knockdown lines showed a significantly diminished ability to learn the relationship between AM and the reward, sucrose (Fig. 2C). These results indicate that *dMpv17* knockdown larvae had a significant learning defect.

Because larvae with impairment of the nervous system often exhibit defects in locomotor behavior^23^, we next evaluated larval locomotor ability by carrying out a crawling assay with wandering-stage larvae. Both knockdown lines showed a significant decrease in average crawling speed (Fig. 2D) and abnormally shortened trajectories (Fig. 2E). In addition to larval locomotor ability, we also examined the locomotor ability of adult flies by performing a climbing assay with *elav*-GAL4. However, no apparent defects in climbing ability were observed (Fig. S1). We next performed a bang-sensitivity assay with adult flies to investigate whether *dMpv17* knockdown induced seizures, but *dMpv17* knockdown flies showed no seizures after exposure to mechanical stress by vortexing (Fig. S2). We also examined the longevity of the neuron-specific *dMpv17* knockdown flies. Although the neuron-specific *dMpv17* knockdown male flies survived longer than elav > GFP-IR flies (Fig. S3), the conclusion has to be waited for further analysis. For some reason, the longevity of the control elav > GFP-IR male flies is much shorter than other control such as Act5C >  + (data not shown).

### *dMpv17* knockdown impairs mitochondrial function in the CNS

**Figure 3:**
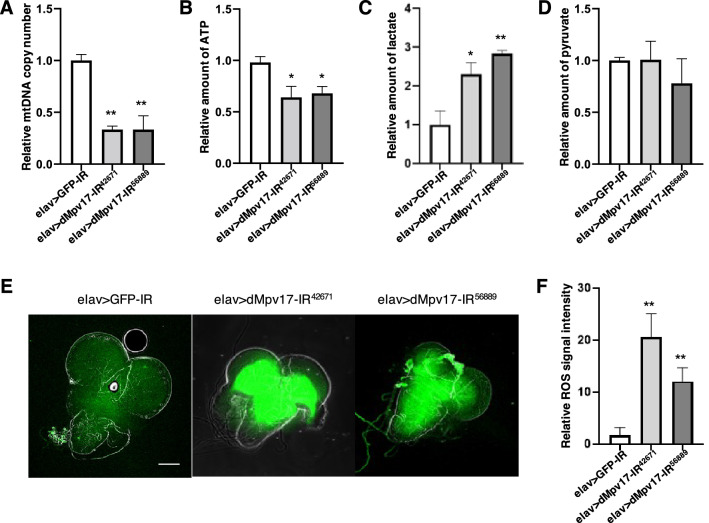
*A dMpv17* knockdown induced mitochondrial dysfunction in the larval CNS. (**A**) The relative mtDNA levels in the larval CNS extract of elav > GFP-IR, elav > dMpv17-IR^42671^, and elav > dMpv17-IR^56889^ larvae were quantified. The relative levels of *16s rRNA* (for mtDNA) and *RpL32* (for nuclear DNA) were measured by qRT-PCR. **P < 0.01, statistical analysis was performed using one-way ANOVA followed by Dunnett’s multiple comparisons test vs elav > GFP-IR. *n* = 3. (**B**) The ATP levels in the larval CNS extracts of elav > GFP-IR, elav > dMpv17-IR^42671^, and elav > dMpv17-IR^56889^ larvae were measured with Cell titer-Glo assay. *P < 0.05, statistical analysis was performed using one-way ANOVA followed by Dunnett’s multiple comparisons test vs elav > GFP-IR. *n* = 5. (**C**) The lactate level in the larval CNS extract of elav > GFP-IR, elav > dMpv17-IR^42671^, and elav > dMpv17-IR^56889^ larvae were measured with the Lactate-Glo assay. **P < 0.01, *P < 0.05, statistical analysis was performed using one-way ANOVA followed by Dunnett’s multiple comparisons test vs elav > GFP-IR. *n* = 3. (**D**) Pyruvate levels in the CNS extracts of elav > GFP-IR, elav > dMpv17-IR^42671^, and elav > dMpv17-IR^56889^ larvae were measured with a pyruvate assay kit. **P < 0.01, *P < 0.05, statistical analysis was performed using one-way ANOVA followed by Dunnett’s multiple comparisons test vs elav > GFP-IR. *n* = 3. (**E**) Third instar larval CNSs were stained with CM-H_2_DCFCA for the detection of ROS. Representative ROS images of the CNS of elav > GFP-IR, elav > dMpv17-IR^42671^, and elav > dMpv17-IR^56889^ are shown. Scale bar, 100 μm. (**F**) The fluorescence intensity in the CNS area enclosed by the white line was quantified. **P < 0.01, statistical analysis was performed using Student’s *t*-test vs elav > GFP-IR. *n* = 5.

Mammalian *Mpv17* encodes an inner mitochondrial membrane protein involved in dNTP uptake^1,2,24^. In addition, in both *MPV17* KO mice and human *MPV17*-related MDDS patients, the mtDNA copy number is significantly reduced^2,10,25^. Therefore, we measured the relative mtDNA copy number in the CNS of neuron-specific *dMpv17* knockdown larvae as indicated by the ratio of mitochondrial-encoded *16s rRNA* to nuclear-encoded *RpL32*. There were statistically significant decreases in relative mtDNA copy number to 22% and 27%, respectively, in elav > dMpv17-IR^42671^ and elav > dMpv17-IR^56889^ compared to the control elav > GFP-IR (Fig. 3A).

Because a decrease in the copy number of mtDNA leads to functional impairment of mitochondria^26,27^, we measured the amount of ATP in the CNS to evaluate the level of mitochondrial activity. Relative ATP levels were reduced to 68% and 65%, respectively, in elav > dMpv17-IR^42671^ and elav > dMpv17-IR^56889^ compared to the control elav > GFP-IR (Fig. 3B). The reduction in ATP levels in neuron-specific *dMpv17* knockdown flies indicates that *dMpv17* knockdown resulted in perturbation of the mitochondria. In addition to the defect in the mitochondria, high levels of reactive oxygen species (ROS) have been detected in the glomeruli and embryonic fibroblasts from *Mpv17*^−/−^ mice^28,29^. We therefore measured ROS in the CNS of *dMpv17* knockdown larvae and found that ROS levels in both knockdown lines were significantly increased (Fig. 3E,F).

Furthermore, in *MPV17*-related MDDS patients, lactic acidosis is often found in addition to the reduction in relative mtDNA copy number^30^. We therefore examined lactate levels in the CNS in both neuron-specific *dMpv17* knockdown fly lines. The relative lactate levels in the CNS increased by 2.3- and 2.8-fold, respectively, in elav > dMpv17-IR^42671^ and elav > dMpv17-IR^56889^ flies compared to the control elav > GFP-IR (Fig. 3C). These data are very similar to symptoms of *MPV17*-related MDDS patients. We also investigated the effect of *dMpv17* knockdown on glycolytic activity by measuring pyruvate levels. However, pyruvate levels were not significantly changed (Fig. 3D). Our findings for lactate and pyruvate levels in the CNS of *dMpv17* knockdown larvae are consistent with findings in HEK293T cells expressing human *MPV17 *carrying the mutation R50W, which was identified in an *MPV17*-related MDDS patient^25^. In addition, the level of ROS production in R50W-expressing cells is greater than that in wild-type *Mpv17*-expressing cells^25^, a result that is broadly consistent with our results for *dMpv17* knockdown larvae.

### Reduction of *dMpv17* induces abnormal morphology of the NMJ

**Figure 4:**
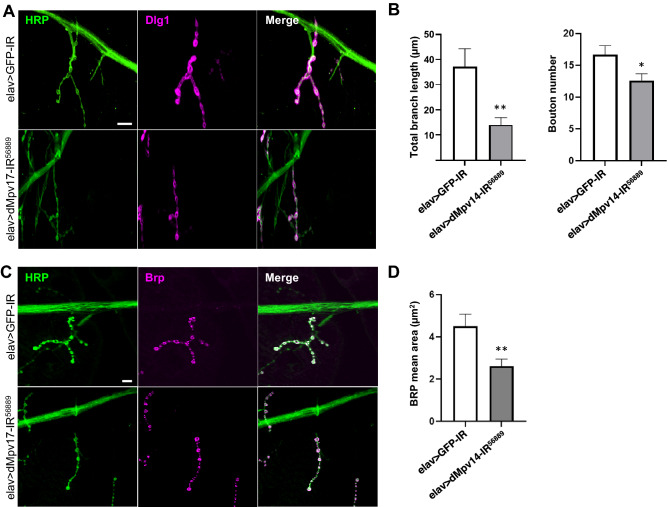
*A dMpv17* knockdown influences larval NMJ morphology. (**A**) Synapses in the NMJ on the fourth muscle of third-instar larvae were inspected in elav > GFP-IR, elav > dMpv17-IR^42671^, and elav > dMpv17-IR^56889^ larvae. The presynaptic terminals were visualized with anti-HRP IgG and the post-synaptic domains with anti-Dlg1 IgG. Scale bar, 10 μm. (**B**) Synapse branch length, bouton numbers were quantified. **P < 0.01, *P < 0.05, statistical analysis was performed using Student’s *t*-test vs elav > GFP-IR. *n* = 15. (**C**) The active zone in the NMJ of the fourth muscle was visualized with anti-HRP and anti-Brp IgGs. Scale bar, 10 μm. (**D**) The area of Brp signals merged with HRP was quantified. **P < 0.01, statistical analysis was performed using Student’s *t*-test vs elav > GFP-IR. *n* = 13.

The defective locomotor ability exhibited by *dMpv17* knockdown larvae may be due to a functional abnormality of neurons. We therefore examined the morphology of neural synaptic terminals at the neuromuscular junctions (NMJs) in third instar larvae. The presynaptic terminals were visualized with the neuronal membrane marker anti-horseradish peroxidase (HRP) IgG and the postsynaptic domains with the postsynaptic marker anti-Discs large 1 (Dlg1) IgG (Fig. 4A). The total length of the synaptic branches, the number of boutons, and the average size of the boutons in the NMJs on the fourth muscle of neuron-specific *dMpv17* knockdown larvae (elav > dMpv17-IR^56889^) were measured and compared with those of control larvae (elav > GFP-IR). While the total length of the synaptic branches and the number of boutons were significantly decreased in *dMpv17* knockdown larvae compared with control (Fig. 4B), the average size of the boutons was not changed (data not shown). At the synaptic terminal there is an active zone where synaptic vesicles fuse to release neurotransmitters into the synaptic cleft and transmit signals to the next synapse or muscle. Therefore, we performed immunostaining with anti-Bruchpilot (Brp) IgG, a marker of the active zone, and measured the density and size of the active zone in the NMJs (Fig. 4C). The area of the active zone of the synapse was reduced in the NMJs of neuron-specific *dMpv17* knockdown larvae (Fig. 4D). These data suggest that the reduction of *dMpv17* leads to the locomotor impairment following the morphological defects in the larval NMJs.

## Discussion

There are ten MPV17/PMP22 family genes in the *Drosophila* genome based on the predicted amino acid sequences. However, only one gene, *Pasang Lhamu*, has been reported to date^31^, and none of the others has yet been characterized. In this study, we demonstrated that *CG11077*, an MPV17/PMP22 family gene, is a *Drosophila* ortholog of mammalian *Mpv17* and named it *dMpv17*. Human *Mpv17* is known as a causal gene of *MPV17*-related hepatocerebral MDDS and CMT^4,6,8^, and models of these diseases have been developed in several organisms such as the mouse and zebrafish^10,17^. However, in spite of the fact that most patients show neurological symptoms, neurological defects have not been investigated in these model organisms.

*Drosophila* is a useful model to study the pathogenic mechanisms of various neurological diseases and disorders^20–22,32^ because the *Drosophila* nervous system is relatively simple, yet it shows good conservation in its basic structure and function with the mammalian nervous system^33,34^. In *Drosophila*, the larval CNS contains 15,000 cells, including 1000 glial cells, and the adult CNS contains 167,000 cells, including 16,000 glial cells^35–37^, while the human brain consists of 100 billion neurons and glial cells^38^. In addition, *Drosophila* larval and adult behavior is less complex than that of mammals but is still complex enough to analyze the effect of changes in gene function. We therefore specifically reduced the expression of *dMpv17* in the *Drosophila* nervous system. Neuron-specific *dMpv17* knockdown larvae showed neurological defects, including locomotor and cognitive impairments. Furthermore, in the larval CNS, the mtDNA copy number and ATP levels were significantly decreased and lactate and ROS levels were increased. These results show good correlation with the symptoms of human *MPV17*-related MDDS and the results obtained in cultured cells derived from patients. In *Mpv17* KO mice, the relative mtDNA copy number is decreased in the liver and muscle but not in the kidney or brain^2,10^. In contrast, neuron-specific *dMpv17* knockdown leads to a reduction in the copy number of mtDNA in the *Drosophila* larval CNS, suggesting that the fly model is more suitable than the mouse model to study the neurological symptoms of human *MPV17*-related MDDS and CMT.

*dMpv17* knockdown induced both locomotor and mitochondrial defects in the larvae like the knockdown larvae of *Solute carrier family 25 member 46a* (*Slc25A46a*) or *Mitochondrial trifunctional protein β subunit* (*Mtpβ/dHADHB*), which are both functional in the mitochondria^39,40^. However, during the adult stage, while the *Slc25A46a* or *Mtpβ/dHADHB* knockdown flies showed locomotor defect, we did not detect any neurological defects, such as those responsible for seizures after severe mechanical stimulus (Fig. S2) or impairment of climbing ability (Fig. S1), even though *dMpv17* is expressed in the adult CNS based on the RNA-seq data in the FlyBase, predicting that *dMpv17* functions in the adult brain. In addition, surprisingly, the climbing ability of elav > GFP-IR was not deceased compared to *dMpv17* knockdown flies throughout adult stage despite the longevity of elav > GFP-IR is significantly shorter (Figs. S1, S3). The observed difference between larvae and adults may be due to inefficient knockdown of *dMpv17* in the adult brain. Because the larval motor neurons in the CNS and PNS are reorganized during metamorphosis^41–45^, if *dMpv17* does not function in adult flies, it might not lead to behavioral impairment. In addition, the other genes which are shown in Fig. 1A might compensate for the *dMpv17* function in adult flies. Alternatively, it might be related to the difference in metabolism between larvae and adult flies. Larvae tend to use more glycolysis because in their short time they need to grow quickly and thus need to use carbons for growth instead of fuel. In contrast, adults primarily use aerobic respiration for their ATP^46–48^. However, further analysis is necessary to clarify this point.

In conclusion, we first showed that *CG11077* is an ortholog of mammalian *Mpv17* and that neuron-specific *CG11077* knockdown induced both locomotor and cognitive defects in *Drosophila* larvae, depending on the mitochondrial dysfunction involved. *dMpv17* knockdown flies showed phenotypes that are very similar to the symptoms of *MPV17*-related hepatocerebral MDDS and CMT caused by *Mpv17* mutation, and at least somewhat similar to the phenotypes of other *Mpv17*^*−/−*^ model organisms, including mouse and zebrafish. Furthermore, it is also interesting whether the oenocyte or fat body, corresponding mammalian liver-specific *dMpv17* knockdown flies mimic the symptoms of *MPV17*-related hepatocerebral MDDS and CMT caused by *Mpv17* mutation. In addition to the similarities of the knockdown phenotypes to the human diseases, *Drosophila* provides various genetic tools to investigate genetically interacting factors and therapeutic candidates relatively easily, making the neuron-specific *dMpv17* knockdown fly a potentially useful model to study the diseases.

## Materials and methods

### Fly stocks

Flies were raised on standard food containing 10% glucose, 5% corn flour, 4% dry yeast, 3% rice bran, and 0.65% agar at 25 °C^49^. White mutant (108479: *w*^*1118*^) and yellow and vermillion double mutant (101249: *y*^*1*^* v*^*1*^) fly strains were sourced from the Kyoto Drosophila Stock Center. UAS-GFP-IR (9331: *w*^*1118*^*; P{w*^+*mC*^ = *UAS-GFP.dsRNA.R}143*), *elav*-GAL4 (8760: w^*^; P{w^+*mC*^ = *GAL4-elav.L}CG16779[3]*), UAS-dMpv17-IR^56889^ (56889: *y*^*1*^ sc^*^ v^*1*^* sev*^*21*^*; P{y[*+ *t7.7] v[*+ *t1.8]* = *TRiP.HMC04110}attP40*) targeting the region corresponding to nucleotide residues 598–618 and 688–708, were sourced from the Bloomington Drosophila Stock Center. UAS-dMpv17-IR^42671^/CyO (42,671: *w*^*1118*^*; P{GD3155}v42671/CyO*) targeting the region corresponding to nucleotide residues 252–577 was obtained from the Vienna Drosophila Resource Center. In order to minimize genetic background effects, the fly lines used in this study were backcrossed several times with the white or yellow vermillion mutant strain.

### Comparison of amino acid sequences of MPV17/PMP22 family proteins

The amino acid sequences of MPV17/PMP family proteins were obtained from UniProt (http://www.uniprot.org). Multiple sequence alignments of protein sequences and the construction of a phylogenetic tree were carried out with the Clustal Omega program^50^.

### Quantitative RT-PCR analysis

RNA was purified from the CNS of male third instar larvae using the RNeasy Lipid Tissue Mini Kit (74804, Qiagen). RNA (100 ng) was used as a template for cDNA synthesis using the PrimeScript RT reagent kit (RR037; Takara). Real-time PCR was performed using SYBR Premix Ex Taq II (RR820; Takara) with 10 ng of cDNA as a template. Specific primers were designed for *dMpv17* (fw 5′-CTTCAATAAGCTGAGCATCG-3′/rev 5′-CGACAATTCTCTGGTTGC-3′) and *glucose-6-phosphate dehydrogenase* (*G6PD*) (fw 5′-AAGCGCCGCAACTCTTTG-3′/rev 5′-AGGGCGGTGTGATCTTCC-3′). PCR was performed using a CFX96 Touch Real-Time PCR Detection System (Bio-Rad), and data were analyzed using CFX Manager Software. The values were read from the calibration curve calculated by CFX Manager Software.

### ROS detection

ROS levels in the larval CNS were measured by the general oxidative stress indicator CM-H_2_DCFDA (C6827, ThermoFisher Scientific). The dissected third instar larval CNSs were shaken in 10 µM CM-H_2_DCFDA in PBS in the dark for 10 min. Then the CNSs were washed with PBS for 5 min and mounted with Vectashield Mounting Medium (Vector Laboratories). Fluorescent patterns were visualized by a confocal laser scanning microscope (Olympus Fluoview FV10i). The fluorescence intensity in the CNS was analyzed by ImageJ software (NIH, USA).

### ATP measurement

ATP levels in the larval CNS were measured by CellTiter-Glo (G7570; Promega) as previously described^51^ with some modifications. Ten instar larval CNSs were homogenized in 100 µL of ATP assay buffer (ab83355; Abcam), and the homogenate was centrifuged at 12,000×*g* at 4 °C for 10 min. The supernatant was then transferred to a new tube, 10 µL of cold trichloroacetic acid (ab204708; Abcam) was added to the supernatant, and the sample was kept on ice for 15 min. After centrifugation at 12,000×*g* for 5 min, the supernatant was transferred to a new tube, and 7.5 µL of neutralization solution (ab204708; Abcam) was added to the supernatant. The sample was incubated on ice for 5 min. In each well of a microtiter plate, 10 µL of sample, 40 µL of ATP assay buffer, and 50 μL of CellTiter-Glo were mixed. Luminescence was read on a Lumat LB 9507 luminometer (Berthold Technologies).

### Lactate measurement

The Lactate-Glo Assay (J5021; Promega) was used to measure lactate levels^52^. After homogenizing ten larval CNSs in 100 µL of PBS, samples were centrifuged at 12,000×*g* at 4 °C for 10 min, and the supernatant was transferred to a new tube. In each well of a microtiter plate, 1 µL of sample, 49 µL of PBS, and 50 µL of lactate detection reagent were mixed and the luminescence was read on a Lumat LB 9507 luminometer.

### Pyruvate measurement

A Pyruvate Assay Kit (MAK071; Sigma) was used to measure pyruvate levels^53^. After homogenizing ten larval CNSs in 100 µL of pyruvate assay buffer, samples were centrifuged at 12,000×*g* at 4 °C for 10 min. The supernatant was then transferred to a new microtiter plate. In each well, 50 µL of sample and 50 µL of pyruvate detection reagent were mixed and the fluorescence was read on Fluoroskan Ascent plate reader (ThermoFisher).

### Climbing assay

Twenty male flies were transferred to a glass vial (9820TST-F25-150, Iwaki) without CO_2_ anesthesia and allowed to habituate for 5 min. The flies were knocked to the bottom by banging the vial twice, then the flies were allowed to climb the wall for 7 s. The height to which each fly climbed was scored as follows: 0 (< 2.0 cm), 1 (2.0–3.9 cm), 2 (4.0–5.9 cm), 3 (6.0–7.9 cm), 4 (8.0–9.9 cm), and 5 (> 10.0 cm). The test was carried out 5 times with a 1-min interval between tests. The climbing index was calculated after arranging the scores of each test in descending order and the averages of the five tests were calculated.

### Bang-sensitivity assay

The bang-sensitivity assay was performed as previously described^54^ with modifications. Seven-day-old flies were transferred to a test vial without CO_2_ anesthesia and allowed to acclimatize for 5 min. The flies were then vortexed at the highest speed for 20 s and were immediately video recorded. The test was repeated after 10 min. The number of flies showing seizure-like responses, characterized by complete immobilization on the back and/or uncontrollable movements for 3 s or more, were counted.

### Crawling assay

Larval locomotion was assessed by crawling assay^23^. Male third instar larvae climbing a wall were used. Assays were performed on 2% agarose gel plates and larval movements were recorded with a video camera for 1 min at a density of 3 larvae per plate. Larval movement distance, length and average speed were analyzed using Image J's wrMTrck plugin.

### Visualization of the neuromuscular junction

To visualize the morphology of the neuromuscular junction (NMJ), male third instar larvae were dissected in HL3 saline solution and fixed in 4% paraformaldehyde in PBS at 25 °C for 30 min. After washing 3 times with PBS containing 0.3% Triton X-100 (PBST) for 10 min, the samples were blocked with PBS containing 0.15% Triton X-100 and 10% normal goat serum for 30 min and incubated with mouse anti-Discs large 1 IgG (4F3; 1:200; DSHB) or mouse anti-Bruchpilot IgG (nc82; 1:200; DSHB) at 4 °C for 16 h. Samples were then washed 3 times with PBST for 10 min and treated for 2 h with anti-mouse IgG labeled with Alexa 594 (A-11032; 1:200; Molecular Probes) and FITC-conjugated goat anti-HRP IgG (1:200; Jackson ImmunoResearch). After washing 3 times with PBST for 10 min, the samples were embedded in Vectashield Mounting Medium and inspected with a confocal laser scanning microscope (Olympus Fluoview FV10i). At the neuromuscular junction, branch length and the numbers of branches and boutons were measured using MetaMorph Microscopy Automation and analyzed using Image Analysis Software (Molecular Devices). Synapses and active zones were observed on the fourth muscle in segments A2 to A6.

### Quantification of mitochondrial DNA copy number

Total DNA was purified from 20 third instar larval CNSs using Mitochondrial DNA Isolation Kit (K280-50; Biovision), omitting the step of separating the genomic and mitochondrial DNA. mtDNA copy number was assessed by qPCR using primers for *16S rRNA* (for mtDNA) and *RpL32* (for nuclear DNA)^44^. Real-time PCR was performed using SYBR Premix Ex Taq II (RR820; Takara) with 10 ng of total DNA as a template. Specific primers were designed for *16S rRNA* (fw 5′-TCGTCCAACCATTCATTCCA-3′/rev 5′-TGGCCGCAGTATTTTGACTG-3′) and *RpL32* (fw 5′-AGGCCCAAGATCGTGAAGAA-3′/rev 5′-TGTGCACCAGGAACTTCTTGAA-3′). PCR was performed using a CFX96 Touch Real-Time PCR Detection System, and data were analyzed using CFX Manager Software. The values were read from the calibration curve calculated by CFX Manager Software.

### Odor-taste learning assay

PCR tubes containing 10 µL of *n*-amyl acetate (AM) diluted 50-fold with liquid paraffin were placed at either end of a 10-mm plate filled with 2% agarose gel containing 2 M sucrose (SUC). Third instar larvae in the food were quickly washed in PBS, placed in the center of the plate, and exposed to AM for 5 min to allow them to learn the relationship between AM and SUC. Next, after washing with PBS, the larvae were transferred onto gel without SUC in which PCR tubes containing 10 µL of 1-octanol (OCT) were placed (25506; Nacalai Tesque), and left for 5 min. The training was carried out 3 times and the larvae were transferred to a SUC-free test plate divided into two compartments by a 1-cm-wide neutral zone drawn in the center. After 3 min, the number of larvae that moved to the OCT or AM side was recorded. The period from conditioning to testing was defined as AM/OCT+. The "+" here represents reward. The learning index (LI) was calculated from the AM and OCT preferences as previously described^55^.

### Lifespan analysis

Adult male flies were raised on the standard food under 12 h light–dark cycles at 28 °C. Flies were transferred to new food every 3 or 4 days and the number of dead flies was counted.

### Data analysis

GraphPad Prism 9.4.1 was used to statistically analyze all results. In the qPCR analysis, ATP, lactate, and ROS measurements, and the crawling, climbing, and learning assays, p-values were also calculated by one-way ANOVA with Dunnett’s multiple comparison test. For immunostaining of NMJs (synapse and active zone), p-values were calculated using the unpaired two-tailed Student’s *t*-test. All data are shown as means ± SEM.

## Supplementary Information

Supplementary Figures.

## Data Availability

All raw data used during the current study are available from the corresponding author on request.

## References

[1] Spinazzola A (2006). MPV17 encodes an inner mitochondrial membrane protein and is mutated in infantile hepatic mitochondrial DNA depletion. Nat. Genet..

[2] Rosa ID (2016). MPV17 loss causes deoxynucleotide insufficiency and slow DNA replication in mitochondria. PLoS Genet..

[3] Moss CF (2017). Aberrant ribonucleotide incorporation and multiple deletions in mitochondrial DNA of the murine MPV17 disease model. Nucleic Acids Res..

[4] El-Hattab AW (2018). *MPV17*-related mitochondrial DNA maintenance defect: New cases and review of clinical, biochemical, and molecular aspects. Hum. Mutat..

[5] Bitting CP, Hanson JA (2016). Navajo neurohepatopathy: A case report and literature review emphasizing clinicopathologic diagnosis. Acta Gastroenterol. Belg..

[6] Baumann M (2019). MPV17 mutations in juvenile- and adult-onset axonal sensorimotor polyneuropathy. Clin. Genet..

[7] Blakely EL (2012). MPV17 mutation causes neuropathy and leukoencephalopathy with multiple mtDNA deletions in muscle. Neuromuscul. Disord..

[8] Choi YR (2015). A novel homozygous MPV17 mutation in two families with axonal sensorimotor polyneuropathy. BMC Neurol..

[9] Weiher H, Noda T, Gray DA, Sharpe AH, Jaenisch R (1990). Transgenic mouse model of kidney disease: Insertional inactivation of ubiquitously expressed gene leads to nephrotic syndrome. Cell.

[10] Viscomi C (2009). Early-onset liver mtDNA depletion and late onset proteinuric nephropathy in *Mpv17* knockout mice. Hum. Mol. Genet..

[11] Meyer zumGottesberge AM, Massing T, Hansen S (2012). Missing mitochondrial Mpv17 gene function induces tissue-specific cell-death pathway in the degenerating inner ear. Cell Tissue Res..

[12] Müller M (1997). Loss of auditory function in transgenic Mpv17-deficient mice. Hear. Res..

[13] Ren JQ, McCarthy WR, Zhang H, Adolph AR, Li L (2002). Behavioral visual responses of wild-type and hypopigmented zebrafish. Vis. Res..

[14] White RM (2008). Transparent adult zebrafish as a tool for in vivo transplantation analysis. Cell Stem Cell.

[15] Krauss J, Astrinides P, Frohnhöfer HG, Walderich B, Nüsslein-Volhard C (2013). *Transparent*, a gene affecting stripe formation in zebrafish, encodes the mitochondrial protein Mpv17 that is required for iridophore survival. Biol. Open..

[16] D’Agati G (2017). A defect in the mitochondrial protein Mpv17 underlies the transparent casper zebrafish. Dev. Biol..

[17] Bian WP (2021). Loss of mpv17 affected early embryonic development via mitochondria dysfunction in zebrafish. Cell Death Discov..

[18] Sang TK, Jackson GR (2005). *Drosophila* models of neurodegenerative diseases. NeuroRx.

[19] Jeibmann A, Paulus W (2009). *Drosophila melanogaster* as a model organism of brain diseases. Int. J. Mol. Sci..

[20] Xiong Y, Yu J (2018). Modeling Parkinson’s disease in *Drosophila*: What have we learned for dominant traits?. Front. Neurol..

[21] Kitani-Morii F, Noto YI (2020). Recent advances in *Drosophila* models of Charcot–Marie–Tooth disease. Int. J. Mol. Sci..

[22] Layalle S, They L, Ourghani S, Raoul C, Soustelle L (2021). Amyotrophic lateral sclerosis genes in *Drosophila melanogaster*. Int. J. Mol. Sci..

[23] Brooks DS, Vishal K, Kawakami J, Bouyain S, Geisbrecht ER (2016). Optimization of wrMTrck to monitor *Drosophila* larval locomotor activity. J. Insect. Physiol..

[24] Trott A, Morano KA (2004). SYM1 is the stress-induced *Saccharomyces cerevisiae* ortholog of the mammalian kidney disease gene *Mpv17* and is required for ethanol metabolism and tolerance during heat shock. Eukaryot. Cell..

[25] Jacinto S (2021). MPV17 mutations are associated with a quiescent energetic metabolic profile. Front. Cell Neurosci..

[26] Holmuhamedov E, Jahangir A, Bienengraeber M, Lewis LD, Terzic A (2003). Deletion of mtDNA disrupts mitochondrial function and structure, but not biogenesis. Mitochondrion.

[27] Fukuoh A (2014). Screen for mitochondrial DNA copy number maintenance genes reveals essential role for ATP synthase. Mol. Syst. Biol..

[28] Antonenkov VD (2015). The human mitochondrial DNA depletion syndrome gene MPV17 encodes a non-selective channel that modulates membrane potential. J. Biol. Chem..

[29] Binder CJ, Weiher H, Exner M, Kerjaschki D (1999). Glomerular overproduction of oxygen radicals in Mpv17 gene-inactivated mice causes podocyte foot process flattening and proteinuria. Am. J. Pathol..

[30] Kim J (2016). MPV17 mutations in patients with hepatocerebral mitochondrial DNA depletion syndrome. Mol. Genet. Metab. Rep..

[31] Jha AR (2016). Shared genetic signals of hypoxia adaptation in *Drosophila* and in high-altitude human populations. Mol. Biol. Evol..

[32] Ugur B, Chen K, Bellen HJ (2016). *Drosophila* tools and assays for the study of human diseases. Dis. Model Mech..

[33] Freeman MR (2015). *Drosophila* central nervous system glia. Cold Spring Harb. Perspect. Biol..

[34] Hunter I, Coulson B, Zarin AA, Baines RA (2021). The *Drosophila* larval locomotor circuit provides a model to understand neural circuit development and function. Front. Neural. Circuits..

[35] Ito K, Urban J, Technau GM (1995). Distribution, classification, and development of *Drosophila* glial cells in the late embryonic and early larval ventral nerve cord. Rouxs Arch. Dev. Biol..

[36] Heckscher ES (2014). Atlas-builder software and the eNeuro atlas: Resources for developmental biology and neuroscience. Development.

[37] Crews ST (2019). *Drosophila* embryonic CNS development: Neurogenesis, gliogenesis, cell fate, and differentiation. Genetics.

[38] von Bartheld CS, Bahney J, Houzel SH (2016). The search for true numbers of neurons and glial cells in the human brain: A review of 150 years of cell counting. J. Comp. Neurol..

[39] Li J (2019). Neuron-specific knockdown of *Drosophila HADHB* induces a shortened lifespan, deficient locomotive ability, abnormal motor neuron terminal morphology and learning disability. Exp. Cell Res..

[40] Ali MS (2020). Neuron-specific knockdown of solute carrier protein SLC25A46a induces locomotive defects, an abnormal neuron terminal morphology, learning disability, and shortened lifespan. IBRO Rep..

[41] Truman JW (1990). Metamorphosis of the central nervous system of *Drosophila*. J. Neurobiol..

[42] Tissot M, Stocker RF (2000). Metamorphosis in *Drosophila* and other insects: The fate of neurons throughout the stages. Prog. Neurobiol..

[43] Choi YJ, Lee G, Park JH (2006). Programmed cell death mechanisms of identifiable peptidergic neurons in *Drosophila melanogaster*. Development.

[44] Winbush A, Weeks JC (2011). Steroid-triggered, cell-autonomous death of a *Drosophila* motoneuron during metamorphosis. Neural Dev..

[45] Subramanian A (2017). Remodeling of peripheral nerve ensheathment during the larval-to-adult transition in *Drosophila*. Dev. Neurobiol..

[46] Tennessen JM, Baker KD, Lam G, Evans J, Thummel CS (2011). The *Drosophila* estrogen-related receptor directs a metabolic switch that supports developmental growth. Cell Metab..

[47] Sen A, Damm VT, Cox RT (2013). Drosophila *clueless* is highly expressed in larval neuroblasts, affects mitochondrial localization and suppresses mitochondrial oxidative damage. PLoS ONE.

[48] Tennessen JM (2014). Coordinated metabolic transitions during *Drosophila* embryogenesis and the onset of aerobic glycolysis. G3.

[49] Kowada R (2021). The function of *Scox* in glial cells is essential for locomotive ability in *Drosophila*. Sci. Rep..

[50] Sievers F (2011). Fast, scalable generation of high-quality protein multiple sequence alignments using Clustal Omega. Mol. Syst. Biol..

[51] Shimizu J (2020). Novel *Drosophila* model for parkinsonism by targeting phosphoglycerate kinase. Neurochem. Int..

[52] Brischigliaro M (2021). Modelling of *BCS1L*-related human mitochondrial disease in *Drosophila melanogaster*. J. Mol. Med..

[53] Ma Z (2018). Epigenetic drift of H3K27me3 in aging links glycolysis to healthy longevity in *Drosophila*. Elife.

[54] Farhan SMK (2017). Identification of a novel synaptic protein, TMTC3, involved in periventricular nodular heterotopia with intellectual disability and epilepsy. Hum. Mol. Genet..

[55] Gerber B, Biernacki R, Thum J (2013). Odor–taste learning assays in *Drosophila* larvae. Cold Spring Harb. Protoc..

