# Supplementary material for: A Drosophila model of the neurological symptoms in Mpv17-related diseases
Source: Sci Rep. 2022 Dec 31;12:22632. doi: 10.1038/s41598-022-27329-x (PMC9805426; doi:10.1038/s41598-022-27329-x)
Supplement: Supplementary file 1 — Supplementary Figures. [file 41598_2022_27329_MOESM1_ESM.docx]

Supplementary Fig. 1


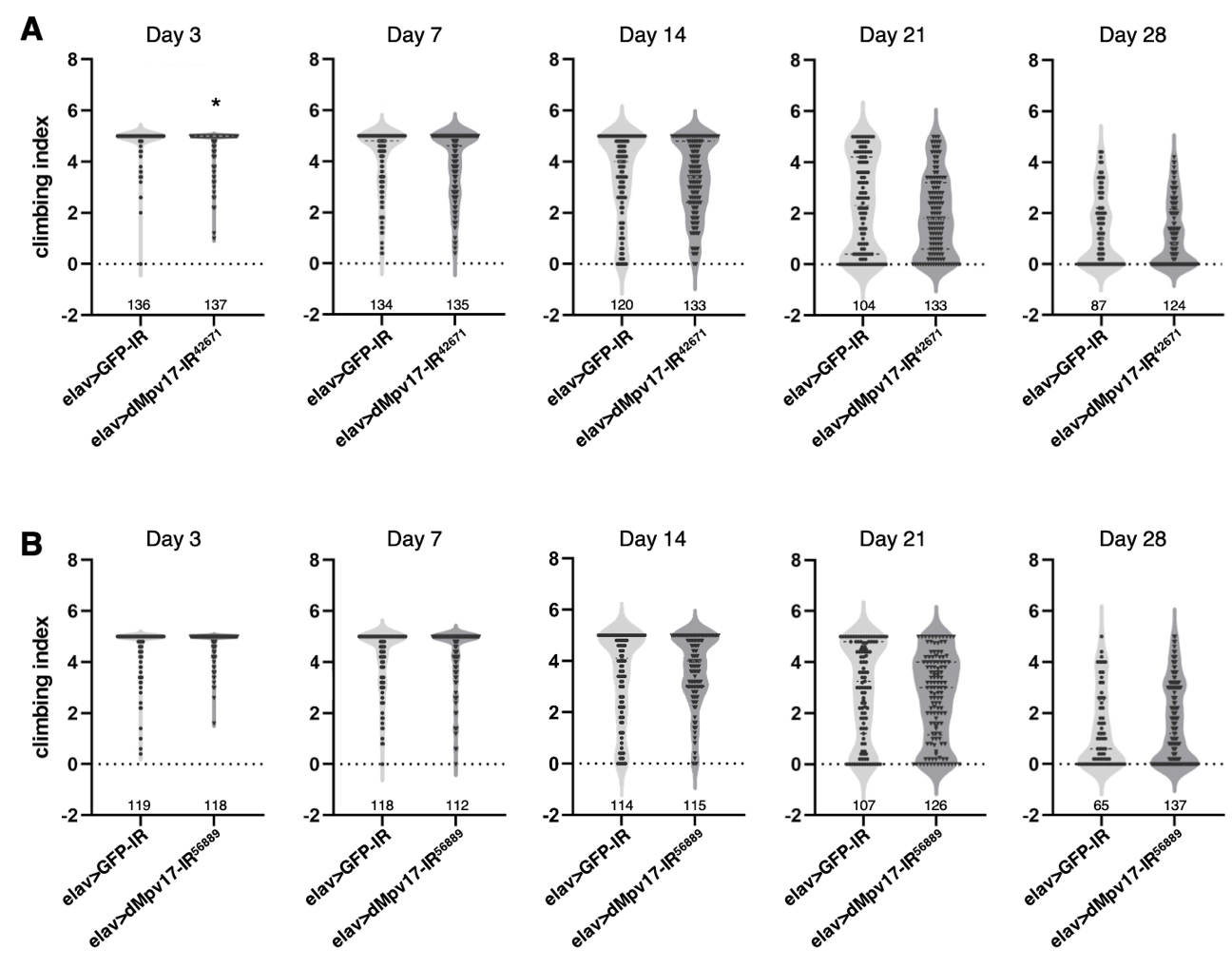


Supplementary Fig. 1 Knockdown of *dMpv17* did not affect climbing ability. (A) The climbing score of elav>dMpv17-IR^42671^ (A) or elav>dMpv17-IR^56889^ (B) on various days after eclosion was compared with that of the control elav>GFP-IR fly. **P*<0.05, statistical analysis was performed using Student’s *t*-test vs elav>GFP-IR.

Supplementary Fig. 2


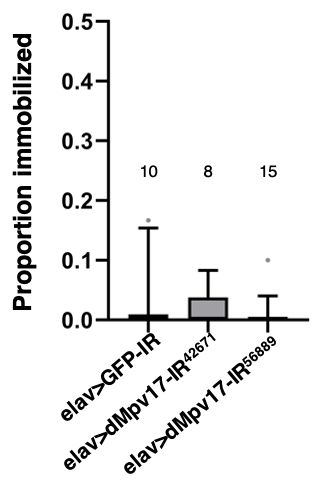


Supplementary Fig. 2 Knockdown of *dMpv17* did not induce seizures. The dot plot shows the proportion of adult flies immobilized for more than 3 s after vortexing at the highest speed. The statistical analysis was performed using one-way ANOVA followed by Dunnett’s multiple comparisons test vs elav>GFP-IR. The number of replicate experiments is shown above each genotype. The bar graph shows the mean ± SEM.

Supplementary Fig. 3


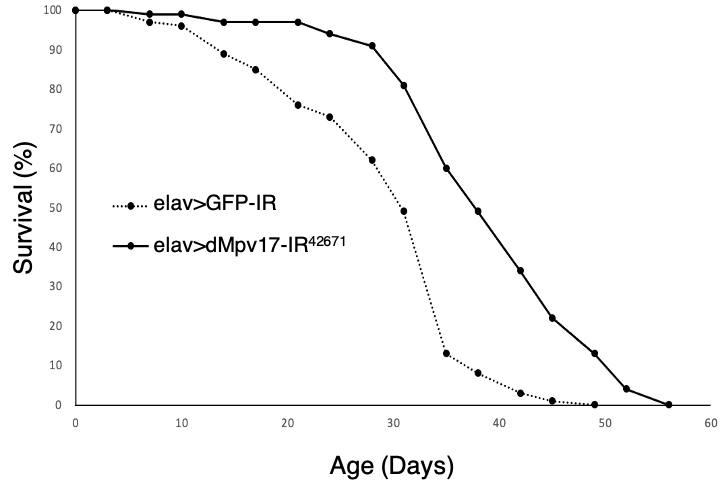


Supplementary Fig. 3 Knockdown of *dMpv17* did not affect the life span of adult male flies. The percentage survival of adult male flies carrying *UAS-GFP-IR/+; elav-GAL4/+* (n=136) or *UAS-dMpv17-IR^42671^/+; elav-GAL4/+* (n=137) The median lifespan for each strain is 31 days and 38 days, respectively.
